# A Retrospective Review of Lead Migration Rate in Patients Permanently Implanted with Percutaneous Leads and a 10 kHz SCS Device

**DOI:** 10.1155/2021/6639801

**Published:** 2021-02-04

**Authors:** Mayank Gupta, Alaa Abd-Elsayed, Meghan Hughes, Anand Rotte

**Affiliations:** ^1^Kansas Pain Management and Neuroscience Research Center, Overland Park, KS, USA; ^2^University of Wisconsin School of Medicine and Public Health, Madisson, WI, USA; ^3^Clinical and Regulatory Affairs, Nevro Corp., Redwood City, CA, USA

## Abstract

**Background:**

Spinal cord stimulation (SCS) has been used over decades for pain management, but migration of percutaneous leads has been the most common complication. Better surgical techniques and newer SCS technologies likely reduced the incidence of lead migration requiring surgical revision, although data are sparse. This study aimed to retrospectively evaluate the incidence of clinically significant percutaneous lead migration in patients permanently implanted with a 10 kHz SCS system.

**Methods:**

Consecutive patients with chronic trunk and/or limb pain, permanently implanted between January 2016 and June 2019, were included in the analysis. Data were collected from the hospital's electronic medical records and the manufacturer's database. Clinically significant lead migration, defined as diminished pain relief followed by surgery to correct lead location, was assessed at the 6-month follow-up.

**Results:**

At the 6-month follow-up, there were no cases of clinically significant lead migration, average pain relief was 65.2%, 82% of patients had response (≥50% pain relief), improvement of function was noted in 72% of patients, and decrease of medication was observed in 42% of patients. Therapy efficacy was sustained in patients with >12 months follow-up; the average pain relief was 58.5%, and the response rate was 82%.

**Conclusions:**

The surgical techniques in use today are designed to minimise the risk of percutaneous lead migration and may have reduced its incidence. In addition, new SCS systems may give greater opportunity to mitigate cases of minor lead movement using alternative stimulation programs.

## 1. Introduction

It is well-established that pain is the leading cause of lost workdays globally [1]. Patients with chronic pain often seek out extensive and expensive medical therapies in an effort to relieve their symptoms. Unfortunately, chronic pain is often difficult to relieve for any substantial period of time, causing a significant impact on quality of life and activities of daily living. Patients with intense, refractory chronic pain often need significant and invasive interventions to produce relief.

In 1967, Shealy and colleagues reported the first case of spinal cord stimulation (SCS), providing successful pain relief in a patient with metastatic cancer [2]. According to the gate control theory of pain postulated by Melzack and Wall in 1965 [3], SCS provides continuous pain relief by stimulating large myelinated fibres in the dorsal column to close the gate to nociceptive pain signals conducted by small unmyelinated nerve fibres [4]. Spinal cord stimulation is used to treat various chronic pain syndromes, including failed back surgery syndrome (FBSS), complex regional pain syndrome, and ischemic pain [5].

The efficacy of SCS is well-established, as is its associated complications: risk of infection, surgery-related pain, and device malfunction [6, 7]. However, one of the most challenging complications to manage and mitigate has been lead migration. In older generations of percutaneous leads, the incidence rates of this technical complication were in the range of 13%–23% [6–8]. Of course, these high rates produced concerns for all parties involved due to the potential requirement for additional corrective surgery.

As the issue of lead migration unfolded, surgical (paddle) leads emerged as possibly providing reduced migration rates compared with percutaneously placed leads [9–12]. However, the landscape of SCS hardware and surgical practice has changed dramatically since these initial studies were carried out, with efforts primarily focused on improving efficacy and reducing complications. Increased awareness of surgical techniques designed to minimise complications (including lead migration) [13], the widespread use of 8-contact leads [14, 15], along with significant strides made in anchoring technology, has reduced the rate of percutaneous lead migration requiring revision in recent years to between 2% and 9% [14–17].

Furthermore, novel waveforms have been developed over the last decade. One such waveform is 10 kHz SCS, which delivers low-amplitude (1.0–5.0 mA) electrical pulses to the spinal cord at 10 kHz frequency and 30 µs pulse width via leads placed in the epidural space of the spinal canal. Unlike during traditional (low-frequency) SCS, whose success is dependent on paraesthesia masking pain in the affected area [18], patients do not experience any paraesthesia during 10 kHz SCS [19]. A high-quality randomised controlled trial (RCT) demonstrated the long-term statistical superiority of 10 kHz SCS over traditional SCS, with 24-month back pain and leg pain responder rates in the 10 kHz SCS group of 77% and 73%, respectively [19, 20]. Other prospective and real-world studies that evaluated 10 kHz SCS for back and leg pain over 12–24 months reported responder rates ranging from 60% to 80% [21–24]. Results from studies that evaluated 10 kHz SCS in pelvic pain, abdominal pain, postsurgical pain, and neck and/or upper limb pain were equally encouraging, with responder rates generally exceeding 80% [25–28]. Studies have also shown that the therapy is associated with improved disability, sleep, and quality of life [29–31]. Furthermore, 10 kHz SCS treatment may facilitate decreased consumption of pain-relieving medications, including opioids [32].

However, evidence of reduced clinically significant lead migration rates with modern implant procedures and novel SCS hardware is sparse [14]. Therefore, we retrospectively analysed data from patients in our centre permanently implanted with percutaneous leads and a 10 kHz SCS device using surgical techniques designed to minimise the risk of lead migration. We present the incidence of clinically relevant lead migration and other outcomes such as response rate, improvements in function, and changes in medication usage.

## 2. Methods

### 2.1. Ethics Statement

Institutional review board (IRB) approval was obtained for the study from Western Institute Review Board. Informed consent was not required and was waived by the IRB due to the retrospective nature of the study and the use of anonymous data.

This study is a retrospective case series of all patients with chronic trunk and/or limb pain who underwent permanent implantation of percutaneous leads and a 10 kHz SCS device (Senza^TM^, Nevro Corp., Redwood City, CA, USA) between January 2016 and June 2019 at the University of Wisconsin Hospital, USA. A single surgeon carried out all procedures. All patients who had failed more conservative treatments were considered appropriate candidates for 10 kHz SCS as part of their standard of care and had undergone a successful therapy trial prior to permanent implantation. Data were gathered from the hospital's electronic medical records (EMRs) and the manufacturer's anonymised commercial database (NevroCloud^TM^).

### 2.2. Procedure

In accordance with our routine clinical practice, all patients with chronic pain who were refractory to conventional treatment and were deemed appropriate candidates for 10 kHz SCS underwent an initial trial for 7 days. If the trial was successful (pain relief ≥50%), patients proceeded to permanent implantation.

**Figure 1:**
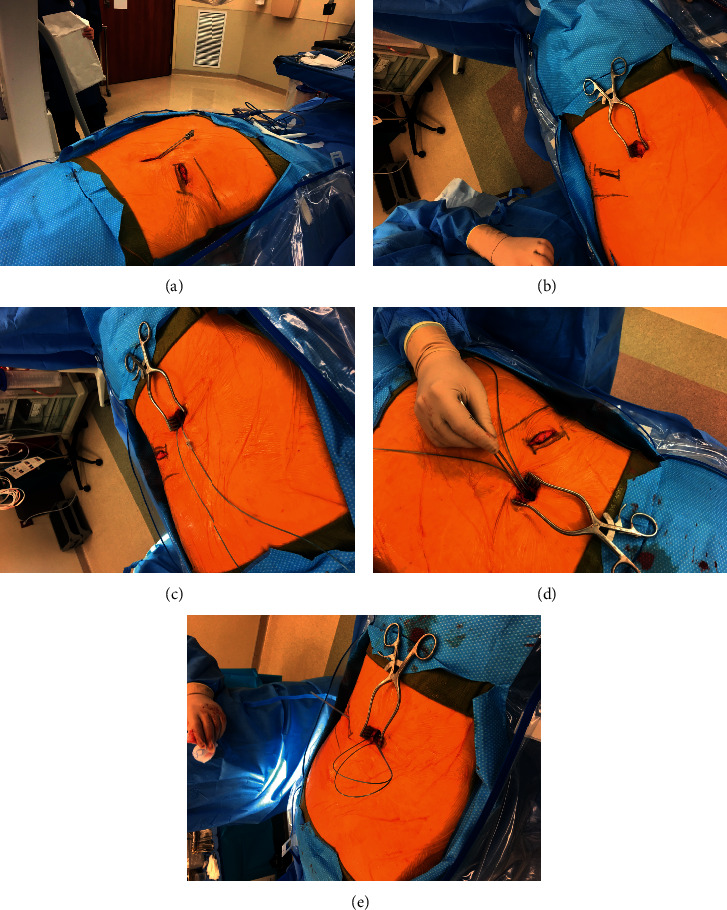
Surgical procedure during trial and implantation of the leads and device.

Under general anaesthesia, a small incision of 1.5–2.0 cm was made approximately 1½–2 vertebral body levels below the intended site of needle entry in the interlaminar space. The incision was dissected down to the level of the lumbodorsal fascia (Figure 1(a)). The needle entry point varied according to the body mass index of the patient to ensure that the angle of entry into the epidural space was 30 degrees or less. The same entry point was used to advance two 14-gauge Touhy needles into the epidural space using the loss of resistance technique (Figure 1(b)).

Two 8-contact stimulation leads were introduced through the Touhy needles under fluoroscopic guidance into the midline dorsal epidural space and navigated to the desired vertebral level, usually spanning T8–T12, and placed in a staggered fashion. The Tuohy needles were removed, and the leads were anchored to the lumbodorsal fascia (Figures 1(c) and 1(d)) using newly designed anchors and 2-0 silk suture (Ethicon LLC, USA). A small stab incision was made at the lead entry site through the fascia so that a small portion of the lead anchor was buried under the fascia. A strain relief loop was created at the midline incision site (Figures 1(e) and 1(f)) before the leads were tunnelled towards the implantable pulse generator (IPG) site, usually located between the iliac crest and the 12th rib, ipsilateral to the incision site. Additional strain relief loops were created in the IPG pocket prior to lead connection. The IPG was programmed to deliver stimulation at 10 kHz frequency, 30 µs pulse width, and an amplitude adjusted to maximise the patient's pain relief.

### 2.3. Outcomes

Data were gathered from the hospital's EMRs and the manufacturer's database (NevroCloud^TM^). The primary measure was the incidence of clinically significant lead migration at 6 ± 1-month follow-up physician assessment and the occurrence of any surgical procedure to revise lead location after permanent implantation. Baseline data collected included demographics, the reason for implantation, and preoperative pain intensity score measured using an 11-point numerical rating scale (NRS; 0 = no pain to 10 = worst possible pain). Patient-reported pain relief obtained from the therapy (0% = no pain relief to 100% = complete pain relief) was recorded at the end of the trial and at the 6-month physician follow-up. Additional information in patients with >12 months follow-up assessment was collected from the manufacturer's database, which included patient-reported pain relief, improvement in function (yes or no), and medication intake (increased, decreased, or unchanged/same).

Clinically significant lead migration was defined as patient-reported diminished pain relief that required revision surgery to correct lead location. Patients who did not achieve satisfactory pain relief with therapy had radiological lead position verification during 6-month follow-up and leads were surgically revised if needed. However, this examination was not routinely performed during follow-up at later visits. Therapy response (defined as ≥50% pain relief from baseline) was evaluated from the patient-reported percentage pain relief documented during the trial, at the 6-month follow-up visit, and at the last visit before data analysis.

### 2.4. Statistical Analysis

Continuous variables such as the pain intensity score and patient-reported percentage pain relief are reported as mean ± standard deviation (SD). Patients with ≥50% pain relief were considered responders. All data were analysed as observed. Data were collected and analysed using Excel^TM^.

## 3. Results

### 3.1. Patient Characteristics

Between January 2016 and June 2019, 101 chronic trunk and/or limb patients underwent permanent implantation of percutaneous leads and a 10 kHz SCS device. The demographics and baseline characteristics of the implanted participants are detailed in Table 1. The median age of the patients was 64 ± 14 years, and the mean pain intensity score at baseline was 7.9 ± 1.4 points (NRS). Most patients reported predominant back/leg/back and leg pain and were refractory to conventional treatment as seen by the median time (12.1 months) between first diagnosis and implant with the 10 kHz SCS device (Table 1). Patients were assessed by their physician at 6 months of follow-up.

To study the outcomes at later follow-up, additional information was collected from the manufacturer's database in patients with follow-up assessment >12 months (*N* = 51). Median follow-up in the patients was 24.4 months, and within the subset, the last follow-up was >24 months in 24 patients (Table 2). Due to the retrospective and nonspecified nature of the data collection, outcome information was not available uniformly in all patients. In patients with follow-up assessment >12 months, pain relief information was available in 49 patients, functional improvement data in 51 patients, and medication change information in 47 patients.

### 3.2. Clinically Relevant Lead Migration

**Figure 2:**
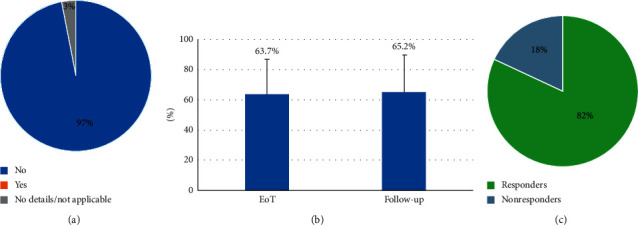
Clinically significant lead migration rate at 6 months of follow-up (a); mean patient-reported pain relief at the end of the trial and the 6-month follow-up visit in 101 patients (b); and responder rate at the 6-month follow-up visit in 101 patients (c).

All cases of patient-reported diminished pain relief at the 6-month follow-up were investigated as potential lead migration incidents. Clinically significant lead migration was defined as diminished pain relief that required revision surgery to correct lead location. Among our cohort, no patients required revision surgery to correct lead location during this 6-month follow-up period (Figure 2(a)).

### 3.3. Pain Relief and Responder Rate

At the end of the trial stimulation, patients reported average pain relief of 63.7 ± 23.2% (Figure 2(b)). At the 6-month physician follow-up visit, average pain relief was maintained, and over 80% of patients (83/101) were responders to therapy (≥50% pain relief; Figure 2(c)). As shown in Table 2, both pain relief and responder rate were further maintained in patients beyond 12 months, and the results were comparable even in patients with follow-up >24 months.

### 3.4. Functional Improvement and Medication

**Figure 3:**
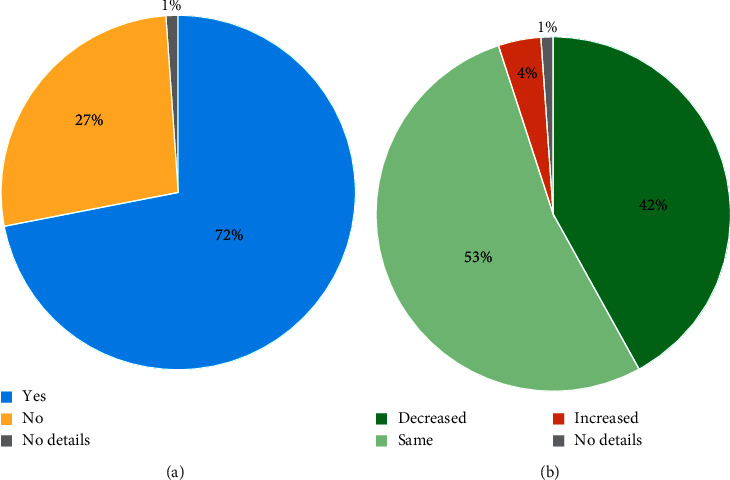
Improvement in function (a) and change in medication (b) at the 6-month follow-up visit (*N* = 101 patients).

**Figure 4:**
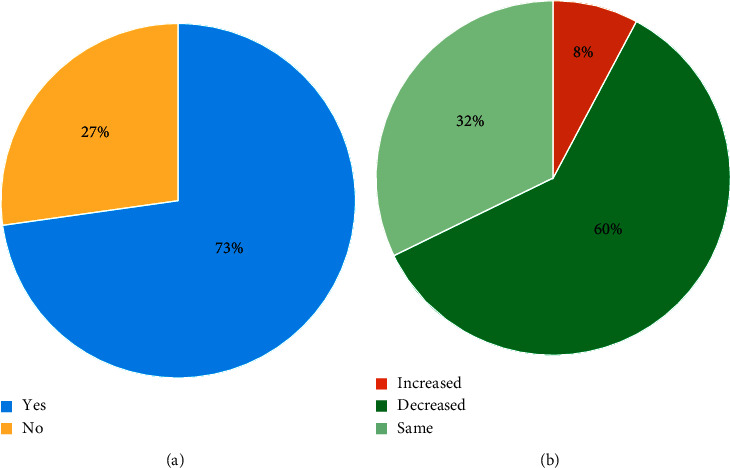
Improvement in function (a) and change in medication (b) in patients with >12 months follow-up (*N* = 51 and 47 patients, respectively).

At the 6-month physician assessment, functional improvement was reported by 72.3% (73/101) of the cohort (Figure 3(a)), and 41.6% (42/101) of patients decreased their medication intake (Figure 3(b)). Further analysis of data from the manufacturer's database was carried out in patients with >12 months follow-up. As shown in Figure 4, functional improvement was sustained over the longer follow-up period, and a higher proportion of patients decreased their medication.

## 4. Discussion

Spinal cord stimulation has been used since 1967 to treat intractable pain, and since that time, it has become an established tool for the treatment of various pain syndromes [33–35]. The safety, efficacy, and cost-effectiveness of SCS have also withstood the test of time [36–44].

As SCS has emerged as front line therapy for patients with chronic neuropathic pain syndromes, one of its greatest issues has been lead migration. Original studies quoted lead migration rates as high as 23% [7]. This technical issue was considered a major limitation due to its purported high incidence, paired with the possible need for surgical correction. In tandem with the migration issue was the emergence of paddle leads being considered superior to their percutaneous counterparts due to lower lead migration rates [9–12]. Only recently have studies been underway to reinvestigate this issue, enabled by the development of new implant techniques and significant technological advancements that have significantly altered the landscape of SCS procedures and outcomes. Our study sought to provide just this type of evidence by providing up-to-date data on clinically significant percutaneous lead migration in patients treated with 10 kHz SCS. Surgical techniques designed to minimise the risk of lead movement were used during implantation.

In our retrospective review of all patients in our centre implanted with percutaneous leads and a 10 kHz SCS device by a single physician over two and a half years, there were no cases of lead migration requiring corrective surgery. Importantly, the responder rate found in our study was similar to rates previously reported for 10 kHz SCS [20], and the improvements in function and medication intake reported by our cohort are in line with other 10 kHz SCS studies [30, 32, 45].

The absence of clinically relevant lead migration among our cohort is important for several reasons. First, it provides additional evidence that the rate of clinically significant percutaneous lead migration has decreased with the development of surgical techniques. It also suggests that device reprogramming can mitigate minor cases of lead movement in 10 kHz SCS systems, potentially reducing the necessity of further interventions to verify electrode position and relocate leads. The absence of paraesthesia in 10 kHz SCS may be beneficial in cases of lead migration that might otherwise produce uncomfortable paraesthesia during postural changes, which is a common occurrence during traditional low-frequency SCS [46, 47]. Furthermore, patients treated with 10 kHz SCS can experience sustained pain relief, quality of life, and disability improvements, as well as reduce their dependence on medication for the management of pain [30–32, 48].

The nil rate of clinically significant lead migration in our cohort is also less than that of the recently published rates for SCS (2%–9%) [14–17]. Overall, the rates are now closer to those recently published for paddle leads (0%–6%) [49–54]. However, the percutaneous approach is less invasive, recovery is faster, and is less costly than paddle lead implantation due to the avoidance of a laminotomy/laminectomy.

The consistent use of surgical techniques designed to reduce the risk of lead migration in our centre may also account for our results. Dissecting all the way through to the lumbodorsal fascia and securing the lead with a modern anchor at this deep fascia level rather than to subcutaneous tissue or muscle may have helped the leads stay in situ [8, 55, 56]. In addition, placing the IPG between the iliac crest and the 12^th^ rib, ipsilateral to the incision site, ensured that the IPG was in the same anatomical plane as the anchor and entry point regardless of body position, thus reducing lead flexion and mobility [57]. Bench tests have shown a 9 cm displacement between the gluteal region and thoracic spine on flexion and extension of the thoracolumbar spine [58]. Hence, traditional placement of the IPG in the buttock area increases the risk of lead migration. Our surgical technique further minimized longitudinal tension on the lead resulting from changes in body position by adding strain relief loops at the midline incision and IPG pocket sites [57]. In addition, we used a shallow needle entry angle of 30 degrees or less during implantation. This approach reduces the risk of fracture and lead migration [59, 60] and allows for more precise lead placement within the epidural space by improving steering capability [56]. Using a needle entry angle of greater than 30 degrees increases tension along the lead, making migration more likely.

### 4.1. Limitations

The main limitation of our study is its retrospective design, which does not take into account all confounders and prevented the collection of X-rays from our cohort that would confirm the presence or absence of lead migration at the follow-up visit after 6-month assessment. As such, our evaluation of clinically significant lead migration was indirect, which limits the interpretation of our results and comparisons with other published literature. Another limitation of our study is the use of patient-reported percentage pain relief instead of a visual analogue scale- (VAS-) based calculation of pain relief, which is a widely used measure. However, VAS scores are not routinely collected during the clinic follow-up, and these data were not retrospectively available for our patients. Finally, all included patients were treated by the same physician; the outcomes are dependent, to some degree, on the expertise and experience of the physician.

## 5. Conclusion

The surgical techniques in use today may have reduced the incidence of clinically significant percutaneous lead migration. In 10 kHz SCS patients, minor cases of lead movement presenting as diminished pain relief may be mitigated with alternative stimulation programs. Since percutaneous lead implantation is less invasive compared with paddle leads, patient recovery can be faster, and the approach could be preferred for permanent implants. Current findings encourage additional studies on cost savings and healthcare utilisation with percutaneous leads versus paddle leads.

## Figures and Tables

**Table 1:** Demographics and clinical characteristics at baseline.

	*N* = 101
Gender	
** **Female	53%
** **Male	47%
Age at implant	64 ± 14 years
Time since diagnosis	12.1 ± 12.5 months
Reason for implant	
FBSS/back surgery/postlaminectomy pain	37%
Radiculopathy	28%
Chronic low back pain	10%
Chronic regional pain syndrome	7%
Feet/leg pain	6%
Chronic postsurgical pain	4%
Other pain	15%
Mean pain intensity score (NRS)	7.9 ± 1.4
Follow-up time for lead migration assessment (months)	6 ± 1

Age and time since diagnosis are presented as median ± SD, and pain intensity score is presented as mean ± SD.

**Table 2:** Pain relief and responder rate in patients with follow-up >12 months.

	All patients	12–24 months follow-up	>24 months follow-up
*N*	49	25	24

Median follow-up time (min, max)	24.4 ± 4.0 months (12.0, 43.3)	16.7 ± 4.0 months (12.0, 23.9)	30.0 ± 4.6 months (24.9, 43.3)

Mean pain relief	58.5 ± 27.7%	63.0 ± 25.0%	53.8 ± 24.0%

Responder rate	82%	88%	75%

Follow-up time is presented as median ± SD, and the pain intensity score is presented as mean ± SD.

## Data Availability

The relevant deidentified data used to support the findings of this study are included within the article. The authors cannot share the raw data from the patients due to privacy concerns.
